# Cordycepin ameliorates cardiac hypertrophy via activating the AMPKα pathway

**DOI:** 10.1111/jcmm.14485

**Published:** 2019-06-21

**Authors:** Hui‐Bo Wang, Ming‐Xia Duan, Man Xu, Si‐Hui Huang, Jun Yang, Jian Yang, Li‐Bo Liu, Rong Huang, Chun‐Xia Wan, Zhen‐Guo Ma, Qing‐Qing Wu, Qi‐Zhu Tang

**Affiliations:** ^1^ Department of Cardiology Renmin Hospital of Wuhan University Wuhan PR China; ^2^ Hubei Key Laboratory of Metabolic and Chronic Diseases Wuhan RP China; ^3^ Department of Cardiology, The First College of Clinical Medical Science China Three Gorges University Yichang PR China; ^4^ Institute of Cardiovascular Diseases China Three Gorges University Yichang PR China

**Keywords:** AMPKα, cardiac hypertrophy, cardiac remodeling, cordycepin, oxidative stress

## Abstract

Increase of myocardial oxidative stress is closely related to the occurrence and development of cardiac hypertrophy. Cordycepin, also known as 3'‐deoxyadenosine, is a natural bioactive substance extracted from *Cordyceps militaris* (which is widely cultivated for commercial use in functional foods and medicine). Since cordycepin suppresses oxidative stress both in vitro and in vivo, we hypothesized that cordycepin would inhibit cardiac hypertrophy by blocking oxidative stress‐dependent related signalling. In our study, a mouse model of cardiac hypertrophy was induced by aortic banding (AB) surgery. Mice were intraperitoneally injected with cordycepin (20 mg/kg/d) or the same volume of vehicle 3 days after‐surgery for 4 weeks. Our data demonstrated that cordycepin prevented cardiac hypertrophy induced by AB, as assessed by haemodynamic parameters analysis and echocardiographic, histological and molecular analyses. Oxidative stress was estimated by detecting superoxide generation, superoxide dismutase (SOD) activity and malondialdehyde levels, and by detecting the protein levels of gp91^phox^ and SOD. Mechanistically, we found that cordycepin activated activated protein kinase α (AMPKα) signalling and attenuated oxidative stress both in vivo in cordycepin‐treated mice and in vitro in cordycepin treated cardiomyocytes. Taken together, the results suggest that cordycepin protects against post‐AB cardiac hypertrophy through activation of the AMPKα pathway, which subsequently attenuates oxidative stress.

## 1 INTRODUCTION

Heart failure (HF), the ultimate outcome of various cardiovascular diseases, is a burgeoning problem that affects more than 20 million people worldwide.^1^, ^2^ Cardiac hypertrophy is an adaptive cellular response to various kinds of biomechanical stresses or overload, that involves increase in the size and/or thickness of cardiac myocytes and the ventricles of the heart. This condition is also associated with enhanced protein synthesis and activation of foetal cardiac gene expression.^3^ Without effective intervention, cardiac hypertrophy is not effectively intervened, eventually develops into HF eventually develops.^4^ Undoubtedly, cardiac hypertrophy plays an important role in the occurrence and development of HF. Pharmacological inhibition of cardiac hypertrophy may be a good way to prevent and treat HF.^5^

Although the exact mechanisms of the cellular responses and pathways associated with cardiac hypertrophy remain unclear, excessive production of reactive oxygen species (ROS) and elevated levels of oxidative stress have recently been identified as conditions that accelerate the hypertrophic process.^6^ Myocardial oxidative stress can be triggered by many factors, including mechanical stretching by pressure overload and various humoral factors, such as angiotensin II (Ang II) and phenylephrine. The signalling pathways closely related to oxidative stress involve oxidative stress included nuclear factor κB (NF‐κB), adenosine 5'‐monophosphate (AMP)‐activated protein kinase α (AMPKα), mitogen activated protein kinases (MAPKs) and other proteins.^7^, ^8^ Among these molecules, AMPKα is important in the regulation of bioenergetic metabolism.^9^ Numerous studies have confirmed that activation of AMPKα can inhibit cardiac hypertrophy and oxidative stress.^9^, ^10^, ^11^ Therefore, searching for a drug that can effectively activate the AMPKα signalling pathway effectively may play a role in inhibiting cardiac hypertrophy.

Cordycepin, also known as 3'‐deoxyadenosine (3'‐dA), is a major natural bioactive substance extracted from *C. militaris*.^12^ *C. militaris* is a valuable medicinal material commonly used in China and other East Asian countries to maintain health and for treatment of diseases involving circulation, respiration, immunity, and the glandular system.^12^ Studies have shown that cordycepin can inhibit airway remodelling in chronically asthmatic rats and inhibit lung fibrosis in cellular and rat models.^13^, ^14^ In addition, cordycepin can activate AMPKα and inhibit oxidative stress.^15^, ^16^, ^17^ However, no research has been performed to elucidate the effect of cordycepin on cardiac hypertrophy. The purpose of this study was to determine whether cordycepin can attenuate cardiac hypertrophy, induced by 1 μmol/L Ang II in cultured neonatal rat cardiac myocytes in vitro and by pressure overload in mice. We also sought to elucidate the molecular mechanism underlying the presumptive effect of cordycepin.

## 2 MATERIALS AND METHODS

### 2.1 Animals

Adult male C57/BL6 mice (8‐10 weeks old, weight: 23.5‐25.5 g) were purchased from the Institute of Laboratory Animal Science, Chinese Academy of Medical Sciences and Peking Union Medical College (CAMS & PUMC; Beijing, China) and housed in the Cardiovascular Research Institute of Wuhan University under controlled temperature and humidity. All animal experimental procedures were conducted in accordance with the Guidelines for the Care and Use of Laboratory Animals of the Chinese Animal Welfare Committee and the guidelines of our hospital, which are consistent with the Guide for the Care and Use of Laboratory Animals published by the United States National Institutes of Health. After 1 week of adaptation, the mice were randomly divided into four groups: a sham operation group (sham, n = 15), a sham + cordycepin treatment group (sham + Cor, n = 15), an aortic banding (AB) group (AB + vehicle, n = 15), and an AB + cordycepin treatment group (AB + Cor, n = 15). Mouse cardiac hypertrophy models were induced by AB. In short, after anaesthetization with pentobarbital sodium by intraperitoneal injection, the left chest of each mouse was opened to identify the thoracic aorta atinin the second intercostal space. We subsequently performed AB operation using 7‐0 silk sutures to band the thoracic aorta against a 27‐gauge needle. The needle was removed and the air was drawn out of the chest before the thoracic cavity was closed. In the sham operation group, similar operations were performed without constricting the aorta. Beginning 7 days after surgery, we administered cordycepin (20 mg/kg/d) by oral gavage for 4 weeks. At the endpoint of the treatment, heart weight/body weight (HW/BW, mg/g), and the heart weight/tibia length (HW/TL, mg/mm) ratios were calculated after the mice were euthanized for both cordycepin and vehicle‐treated mice.

### 2.2 Antibodies and reagents

Cordycepin specified to be over 99.2% pure as determined by HPLC was purchased from Shanghai Winherb Medical Co. (Shanghai, China). Primary antibodies against the following proteins were obtained from Cell Signalling Technology (Danvers, MA): anti‐AMPKα (#2603P), anti‐phospho‐AMPKα (#2535), anti‐mammalian target of rapamycin (mTOR) (#2983), anti‐phospho‐ mTOR (#2971), anti‐phospho‐ERK, #4370P), anti‐ERK (#4695), anti‐acetyl‐CoA carboxylase (ACC) (#3676), anti‐phospho‐ACC (#3661), and anti‐GAPDH (#2118). Primary antibodies against gp^91^phox (ab129068), superoxide dismutase 1 (SOD1, ab16831), SOD2 (ab38155), and α‐actinin (ab68167) were purchased from Abcam (Cambridge, UK). The secondary antibody was purchased from LI‐COR Biosciences. Ang II (A9525) and compound C (CpC, P5499) were purchased from Sigma‐Aldrich. Proteins were measured with assay kits obtained from Pierce (Pierce, 23225).

### 2.3 Echocardiography and haemodynamics

Mice were anaesthetized by inhalation of 1.5% isoflurane, and echocardiography was performed to evaluate the structure and function of the left ventricle using a MyLab system (Esaote SpA, Genoa, Italy) equipped with a 10‐MHz probe. Parasternal short axis images at the mid‐papillary muscle level were recorded in M‐mode. The left ventricular (LV) dimensions, including LV end‐systolic diameter (LVEDs), LV end‐diastolic diameter (LVEDd) and posterior wall thickness were measured and averaged from ten consecutive cardiac cycles. Based on these data, the LV ejection fraction (EF) and fractional shortening (FS) were calculated.

After echocardiography, haemodynamic parameters were measured. The mice were anaesthetized (1.5% isoflurane) sequentially. A 1.4‐French microtip catheter transducer (SPR‐839; Millar Instruments, TX) was inserted into the right carotid artery and advanced into the left ventricle. The heart rate and pressure signals were recorded continuously with a Millar Pressure‐Volume System (Millar Instruments, USA). We selected a stable section of the pressure volume curve and analysed it with PVAN data analysis software.

### 2.4 Morphological analysis

Cardiac tissue samples were washed in 10% potassium chloride solution, and fixed in 10% buffered formalin immediately. After transversely cutting the bottom, the hearts were embedded with paraffin. Several sections of the heart (5 μm thick) were prepared, stained with haematoxylin and eosin (HE) for histopathology or with picrosirius red (PSR) for assessment of interstitial fibrosis, and then visualized by light microscopy. Single myocytes (between 150 and 200 LV myocytes were outlined in each sample, a total of 6 sample) and the LV collagen volume fraction (calculated from the PSR‐stained sections as the area stained by PSR divided by the total area) were measured using a quantitative digital image analysis system (Image‐Pro Plus, IPP, version 6.0).

### 2.5 Cultured neonatal rat ventricular myocytes

Neonatal rat ventricular myocytes (NRVMs) were isolated from Sprague‐Dawley rats within 3 days of birth and cultured as previously described.^18^, ^19^, ^20^ Briefly, the hearts were quickly removed a; then, they were washed with phosphate‐buffered saline (PBS) three times, and incubated with 0.125% trypsin‐EDTA (Gibco, 2520‐072) for 15 minutes at 34°C for a total of five times. Subsequently, the NRVMs were centrifuged via a differential attachment technique then seeded in six‐well culture plates at a density of 2 × 10^5^ cells per well. The isolated NRVMs were grown in DMEM/F12 containing 15% foetal bovine serum (FBS) (Gibco, 10099), 100 mg/mL streptomycin (Gibco, 15140), and 100 U/mL penicillin at 37°C in a humidified incubator with 5% CO_2_. Bromodeoxyuridine (BrdU; 0.1 mmol/L) was used to prevent the growth of cardiac fibroblasts. Before treatment with Ang II (1 μmol/L) and cordycepin (0, 2.5, 5, 10, or 20 μmol/L), the cells were cultured in 1% FBS for 12 hours.

### 2.6 Cell viability

Cell viability was assessed with a Cell Counting Kit‐8 (CCK8) assay (Dojindo, GB707, Japan). After the cells were treated with graded concentrations of cordycepin for 24,48, and 72 hours, 10 μL of CCK8 solution was added to each well of a 96‐well plate and the absorbance was measured at 450 nm by an ELISA reader (SynergyHT, Bio Tek) after 4 hours of incubation according to the manufacturer's instructions. The effect of cordycepin with different concentrations on cell viability were determined by calculating the cell viability percentage of cordycepin‐treated cells compared with that of vehicle‐treated cells (set at 100%).

### 2.7 Immunofluorescence staining

Neonatal rat ventricular myocytes were analysed for cardiac α‐actinin expression by immunofluorescence to assess cardiomyocyte hypertrophy. The cells were washed three times with PBS, fixed with 4% paraformaldehyde, permeabilized in 0.2% Triton™ X‐100 in PBS, and blocked with 8% goat serum for 1 hour at room temperature. Then, the cells were stained with a monoclonal anti‐α‐actinin antibody at a dilution of 1:100 in 1% goat serum overnight. The cells were then incubated with an Alexa Fluor 488‐conjugated goat anti‑rabbit immunoglobulin G (IgG) (Invitrogen, USA) secondary antibody for 1 hour at 37°C. DAPI was used for visualization of nuclei. Images were obtained with a fluorescence microscope (Olympus DX51, Japan). Single myocytes was measured using IPP 6.0. Quantitative data for myocyte size were obtained from >150 randomly selected myocytes in three independent experiments.

### 2.8 Determination of oxidative stress

Superoxide generation and the levels of malondialdehyde (MDA) and SOD, were measured in homogenized cardiac tissue samples using commercially available kits (Nanjing Jiancheng Bioengineering Institute, China) according to the manufacturer's protocol.^21^ The leavel of glutathione peroxidase (GSH‐Px) was detected using an assay kit (Beyotime, China) according to the manufacturer's protocol. The levels of superoxide generation rate was detected using an assay kit (Solarbio, China). The general steps are as follows: the samples were homogenized and the supernatant was centrifuged. According to the instructions, the standard products were prepared and the corresponding reagents were added. Measuring 530 nm absorbance, drawing standard curve and calculating rate.

### 2.9 Quantitative real‐time polymerase chain reaction (RT‐PCR)

Total RNA was extracted from pulverized and homogenized LV tissue using TRIzol reagent (Invitrogen, 15596‐026) and reverse transcribed into cDNA (synthesized from 2 μg of RNA from each group) with an Advantage RT‐for‐PCR Kit (No. 04896866001; Roche, Germany) according to the manufacturer's instructions. Quantitative RT‐PCR analysis was performed with LightCycler 480 SYBR Green 1 Master Mix (No. 04707516001; Roche, Germany) according to the manufacturer's instructions. All primer information was provided in Table 1 and the results were normalized against glyceraldehyde‐3‐phosphate dehydrogenase (GAPDH) expression.

**Table 1:** The primers sequences for RT‐PCR

Targets		Sequence (5'‐3')
Mouse‐GAPDH	Forward	TCATCAACGGGAAGCCCATC
	Reverse	CTCGTGGTTCACACCCATCA
Mouse‐ANP	Forward	ACCTGCTAGACCACCTGGAG
	Reverse	CCTTGGCTGTTATCTTCGGTACCGG
Mouse‐BNP	Forward	GAGGTCACTCCTATCCTCTGG
	Reverse	GCCATTTCCTCCGACTTTTCTC
Mouse‐β‐MHC	Forward	CCGAGTCCCAGGTCAACAA
	Reverse	CTTCACGGGCACCCTTGGA
Mouse‐TGFβ	Forward	GGTGGTATACTGAGACACCTTG
	Reverse	CCCAAGGAAAGGTAGGTGATAG
Mouse‐Collagen1a	Forward	AGGCTTCAGTGGTTTGGATG
	Reverse	CACCAACAGCACCATCGTTA
Mice‐Collagen III	Forward	AAGGCTGCAAGATGGATGCT
	Reverse	GTGCTTACGTGGGACAGTCA
Mice‐αSMA	Forward	GTCCCAGACATCAGGGAGTAA
	Reverse	TCGGATACTTCAGCGTCAGGA
Rat‐GAPDH	Forward	GACATGCCGCCTGGAGAAAC
	Reverse	AGCCCAGGATGCCCTTTAGT
Rat‐ANP	Forward	AAAGCAAACTGAGGGCTCTGCTCG
	Reverse	TTCGGTACCGGAAGCTGTTGCA
Rat‐BNP	Forward	CAGCAGCTTCTGCATCGTGGAT
	Reverse	TTCCTTAATCTGTCGCCGCTGG
Rat‐β‐MHC	Forward	TCTGGACAGCTCCCCATTCT
	Reverse	CAAGGCTAACCTGGAGAAGATG

### 2.10 Western blotting

Total protein was extracted from heart tissue and H9c2 cells were lysed using a RIPA buffer. The protein concentrations were determined using a BCA kit (Thermo Fisher Scientific, USA). The protein concentration of each sample was normalized according to the concentration of each sample detected by the enzyme labelling apparatus. Fifty micrograms of protein lysate was subjected to SDS‐PAGE, and the separated proteins were transferred to polyvinylidene fluoride (PVDF) membranes (EMD Millipore, FL00010, USA) as previously described. After blocking for 1‐2 hours with 5% non‐fat milk at room temperature, the PVDF membranes were incubated with the corresponding primary antibodies overnight at 4°C before being incubated with goat anti‐mouse IgG (LI‐COR, C11026‐03) secondary antibodies for 1 hour at room temperature. The western blot bands were scanned by a two‐colour infrared imaging system (Odyssey, LI‐COR, USA). For quantification, the specific protein expression levels were normalized to GAPDH levels.

### 2.11 Statistical analysis

The data are expressed as the means ± SEM. GraphPad Prism 5.0 software (GraphPad Software, USA) for Windows was used for the analysis. Differences among groups were tested by one way ANOVA followed by the post hoc Tukey test, whereas differences between two groups were compared using unpaired Student's *t* tests. Statistical significance was defined as *P* < 0.05.

## 3 RESULTS

### 3.1 Cordycepin attenuated cardiac hypertrophy induced by AB in vivo

**Figure 1:**
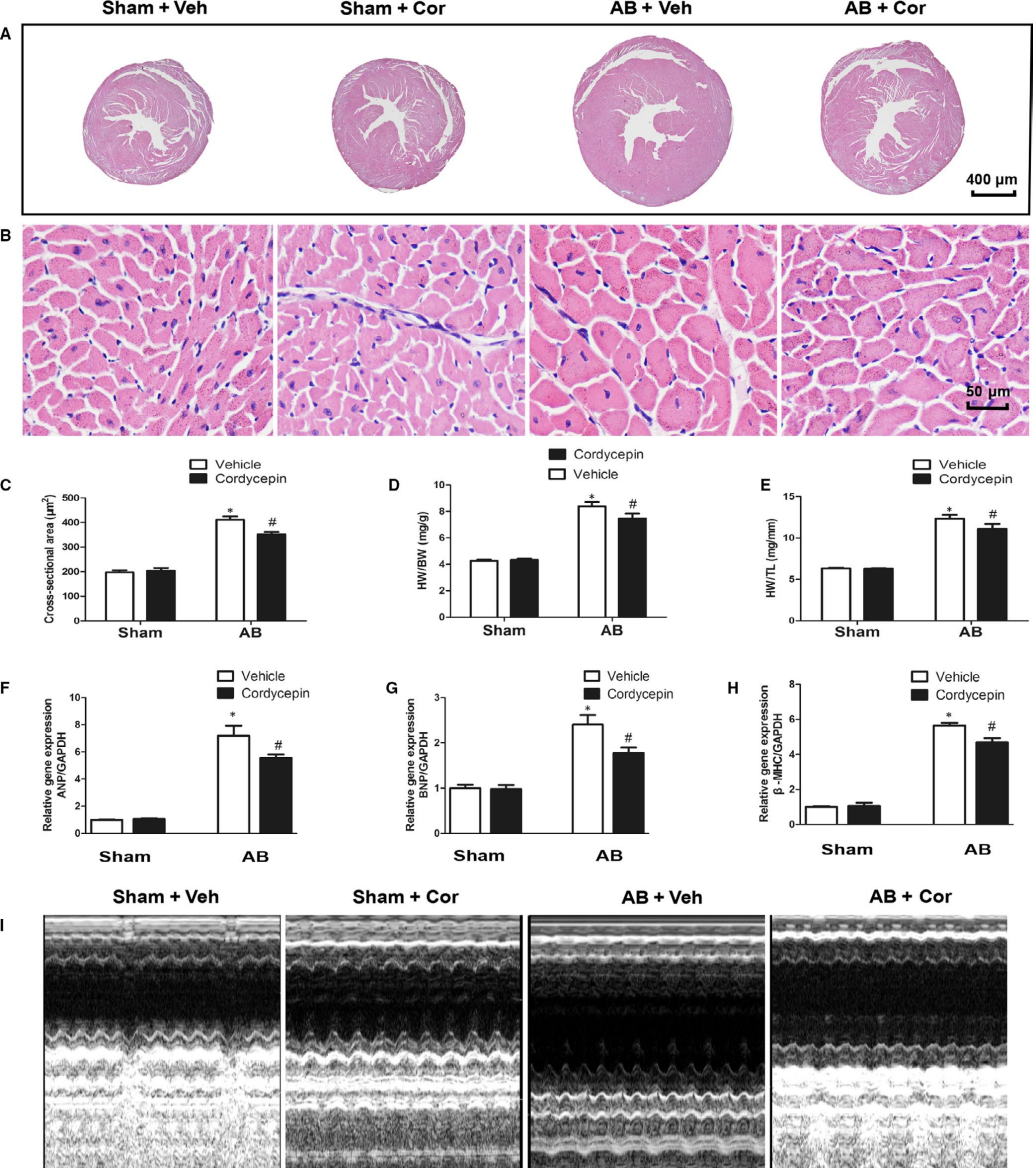
Cordycepin attenuated cardiac hypertrophy induced by pressure‐overload in vivo. (A,B) HE staining of tissues from sham and AB model mice at 4 wk after surgery. The mice were treated with vehicle or cordycepin (n = 6). A: 10×; B: 400×. (C) Statistical results for the cross‐sectional area (n = 6 sample, 150‐200 cells per sample). (D‐E) Statistical results for the HW/BW ratio and HW/TL ratio at 4 wk after AB surgery (n = 10). (F‐H) mRNA levels of hypertrophic markers (n = 6). (I) Echocardiographic representative pictures. ^#^ *P* < 0.05 vs the sham group; *P < 0.05 vs the AB + vehicle group

To determine whether cordycepin antagonized the hypertrophic response to pressure overload, mice were subjected to either AB or sham surgery. AB surgery resulted in a significant increase in the hypertrophic response in vehicle‐treated mice, as estimated by the heart weight to body weight (HW/BW, mg/g) and heart weight to tibia length (HW/TL, mg/mm) ratios, cross‐sectional area (CSA) and foetal gene (atrial natriuretic peptide, ANP; B‐type natriuretic peptide, BNP; and β‐myosin heavy chain, β‐MHC) expression levels, shown in Figure 1F‐H), without affecting body weight or heart rate (Table 2). These changes were significantly attenuated in cordycepin‐treated AB mice (Figure 1A‐H). Furthermore, cordycepin‐treated mice exhibited markedly increased LV EF and LV FS (Table 2), with representative pictures shown in Figure 1I; In addition, analysis of haemodynamic parameters showed that cordycepin treatment improved systolic (assessed by +dP/dt) and diastolic function (assessed by −dP/dt) after AB surgery (Table 2). Collectively, these data suggest that cordycepin ameliorates cardiac hypertrophy and improves cardiac function after pressure overload.

**Table 2:** Echocardiographic and hemodynamic parameters in mice after AB surgery

Parameter	Sham		AB	
	Vehicle	Cordycepin	Vehicle	Cordycepin
LVEDd (mm)	3.63 ± 0.22	3.62 ± 0.22	4.74 ± 0.24^#^	4.3 ± 0.38^#^ ^,^ *
LVESd (mm)	2.23 ± 0.36	2.32 ± 0.36	4.16 ± 0.74^#^	3.44 ± 0.41^#^ ^,^ *
EF (%)	75 ± 7.68	72 ± 9.35	46.92 ± 2.96^#^	55.25 ± 5.96^#^ ^,^ *
FS (%)	38.83 ± 6.62	36.5 ± 7.53	20.17 ± 1.59^#^	26.5 ± 3.23^#^ ^,^ *
HR (min^−1^)	483.93 ± 32.71	490.46 ± 37.71	496.19 ± 38.39	491.75 ± 39.4
ESP (mm Hg)	105.51 ± 3.28	106.47 ± 3.57	154.2 ± 9.84^#^	153.26 ± 12.15^#^
EDP (mm Hg)	11.69 ± 1.28	11.1 ± 1.26	23.45 ± 2.3^#^	22.13 ± 2.64^#^
CO (μL/min)	7869 ± 188	7976 ± 156	4040 ± 284^#^	4602 ± 360^#^ ^,^ *
dP/dt max (mm Hg/s)	8201 ± 393	8374 ± 386	5231 ± 469^#^	6079 ± 432^#^ ^,^ *
dP/dt min (mm Hg/s)	−8128 ± 396	−8019 ± 319	−4776 ± 225^#^	−5240 ± 318^#^

Abbreviations: CO, cardiac output; dp/dtmax, maximal rate of pressure development; dp/dtmin, maximal rate of pressure decay; EDP, end‐diastolic pressure; EF, left ventricular ejection fraction; ESP, end‐systolic pressure; FS, left ventricular fractional shortening; LVEDd, left ventricular end‐diastolic diameter; LVESd, left ventricular end‐systolic diameter.

^#^ *P* < 0.05 for difference from corresponding sham group.

* *P* < 0.05 vs the AB + vehicle group. n = 12.

### 3.2 Cordycepin attenuated cardiac fibrosis in vivo

**Figure 2:**
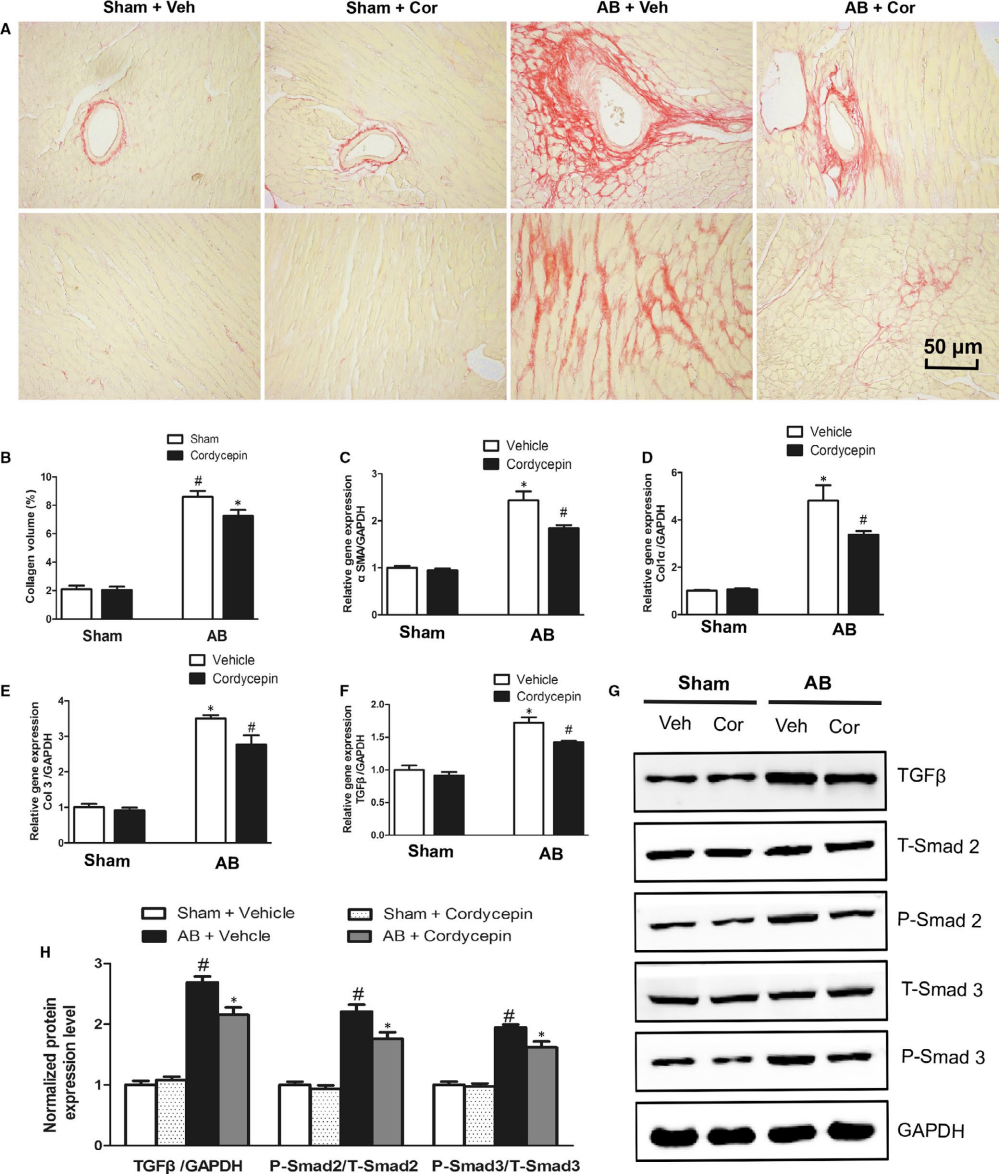
Cordycepin attenuated cardiac fibrosis induced by pressure‐overload in vivo. (A‐C) Representative picrosirius red staining of histological sections and the statistical results (n = 6). (C‐F) Real‐time PCR analysis of fibrosis‐related genes (n = 6). (G,H) Representative blots of TGF‐β1, phosphorylated Smad2, total Smad2, phosphorylated Smad3 and total Smad3 from the indicated groups (n = 5). ^#^ *P* < 0.05 vs the sham group; *P < 0.05 vs the AB + vehicle group.

Cardiac interstitial fibrosis is one of the main features of cardiac hypertrophy and was evaluated by PSR staining and fibrotic marker detection. Cardiac perivascular and interstitial fibrosis was detected in both vehicle‐treated and cordycepin‐treated mice. The sham‐operated mice had very little collagen deposition examined by PSR staining (either in the vehicle‐sham group or in the cordycepin‐sham group), while the collagen deposition increased significantly after AB. Mice with cordycepin‐treated presented a marked reduction in LV collagen volume examined by PSR staining after AB surgery (Figure 2A,B). Subsequent analysis of the mRNA and protein expression levels of known mediators of fibrosis, including transforming growth factor β1 (TGF‐β1) and phosphorylated Smad 2/3. After AB surgery, their mRNA and protein levels were significantly upregulated, but cordycepin treatment could inhibit these fibrosis indicators to some extent. In addition, the mRNA levels of collagen I, collagen III, and α‐SMA were markedly increased in the vehicle treated mice after AB surgery (Figure 2C‐H). However, these changes were alleviated by cordycepin treatment. These results suggest that cordycepin can suppress cardiac interstitial fibrosis after AB surgery.

### 3.3 Cordycepin inhibits oxidative stress in vivo

**Figure 3:**
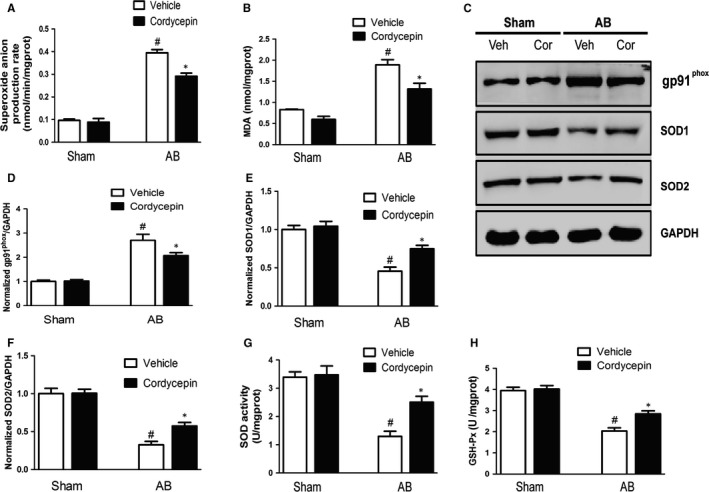
Cordycepin attenuated oxidative stress induced by pressure‐overload in vivo. (A,B) Detection of superoxide anion production rate and malondialdehyde (MDA) generation with related kits. (C‐F) Representative blots of gp91^phox^, SOD1 and SOD2 from indicated groups (n = 5). (G,H) Detection of SOD activity and glutathione peroxidase (GSH‐Px) by related kits. ^#^ *P* < 0.05 vs the sham group; *P < 0.05 vs the AB + vehicle group

Increasing numbers of studies have suggested that oxidative stress promotes cardiac hypertrophy; therefore, to further explore the potential mechanisms contributing to the protective effect of cordycepin, we subsequently measured the levels of protein markers associated with oxidative stress. Figure 3 demonstrates that cardiac superoxide generation, and MDA, and gp91^phox^ (also known as NOX2; a key component of the enzyme NADPH oxidase, which plays a critical role in ROS generation) levels were significantly higher in the AB + vehicle group than in the sham group; however, such elevation was significantly attenuated by treatment with cordycepin (Figure 3A‐D). Conversely, the levels and activity of SOD (an antioxidative enzyme), and the levels of glutathione peroxidase (GSH‐Px) were notably decreased in the AB model‐vehicle group than in the sham group, an effect that was significantly reversed by cordycepin (Figure 3E‐H).

### 3.4 Cordycepin promoted AMPKα phosphorylation in hypertrophic hearts

**Figure 4:**
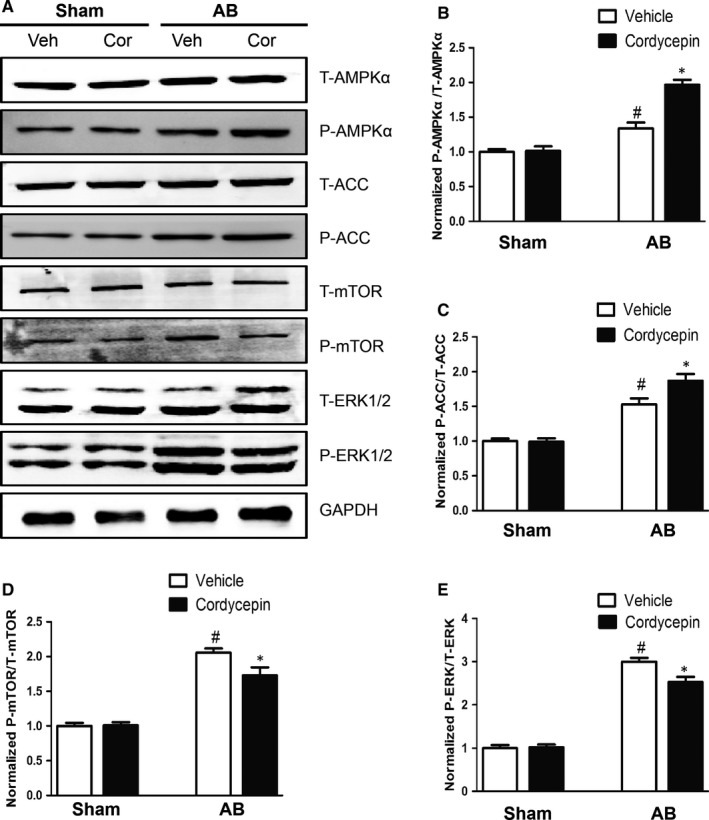
Effects of cordycepin on the AMPKα, ACC, mTOR and ERK1/2 signalling pathway. (A‐E) Protein levels of phosphorylated AMPKα and related targets in the indicated groups (n = 5). ^#^ *P* < 0.05 vs the sham group; *P < 0.05 vs the AB + vehicle group

To investigate the molecular mechanism by which cordycepin ameliorated cardiac hypertrophy, we examined the effects of cordycepin on AMPKα and its downstream signalling pathway. Phosphorylated ACC is a substrate of AMPKα, and its levels can thus reflect the activity of AMPKα. Our research confirm that phosphorylated AMPKα and ACC were elevated in AB + Cor mice compared to AB model mice. In addition, phosphorylation of mTOR and ERK1/2 was induced by AB. The mTOR and ERK1/2 signalling pathway is an important mediator of cardiac hypertrophy, and the observed changes in this pathway were alleviated by cordycepin treatment (Figure 4).

### 3.5 Cordycepin attenuated Ang II induced cardiomyocyte hypertrophy

To determine the possible cytotoxicity of cordycepin towards cardiomyocytes, we evaluated cell viability using a CCK8 assay. NRVMs were incubated with various concentrations of cordycepin (0, 2.5, 5, 10, and 20 μmol/L) for 24, 48 and 72 hours. Cardiomyocyte viability was not affected at each time point (24, 48, 72 hours) by cordycepin at concentrations from 0 to 20 µmol/L (Figure S1A). An in vitro model, NRVMs were cultured with 1 μmol/L Ang II for 24 hours to confirm the effects of cordycepin on cardiac hypertrophy. Cardiomyocytes were pre‐incubated with cordycepin (0, 2.5, 5, 10, or 20 μmol/L) for 1 hour and then treated with 1 μmol/L Ang II. The Ang II‐treated model NRVMs showed greater cell surface areas than those both treated with cordycepin (5, 10 and 20 μmol/L) and stimulated with Ang II (Figure S1B,C). Further experiments showed that cordycepin (5, 10 and 20 μmol/L) inhibited the activity of ANP and BNP and the β‐MHC promoter (Figure S1D‐F). The results showed that cordycepin could significantly inhibit cardiomyocyte hypertrophy in vitro as well as in vivo. The 20 μmol/L cordycepin concentration was selected for further study.

### 3.6 CpC counteracted the protective effects of cordycepin in vitro

**Figure 5:**
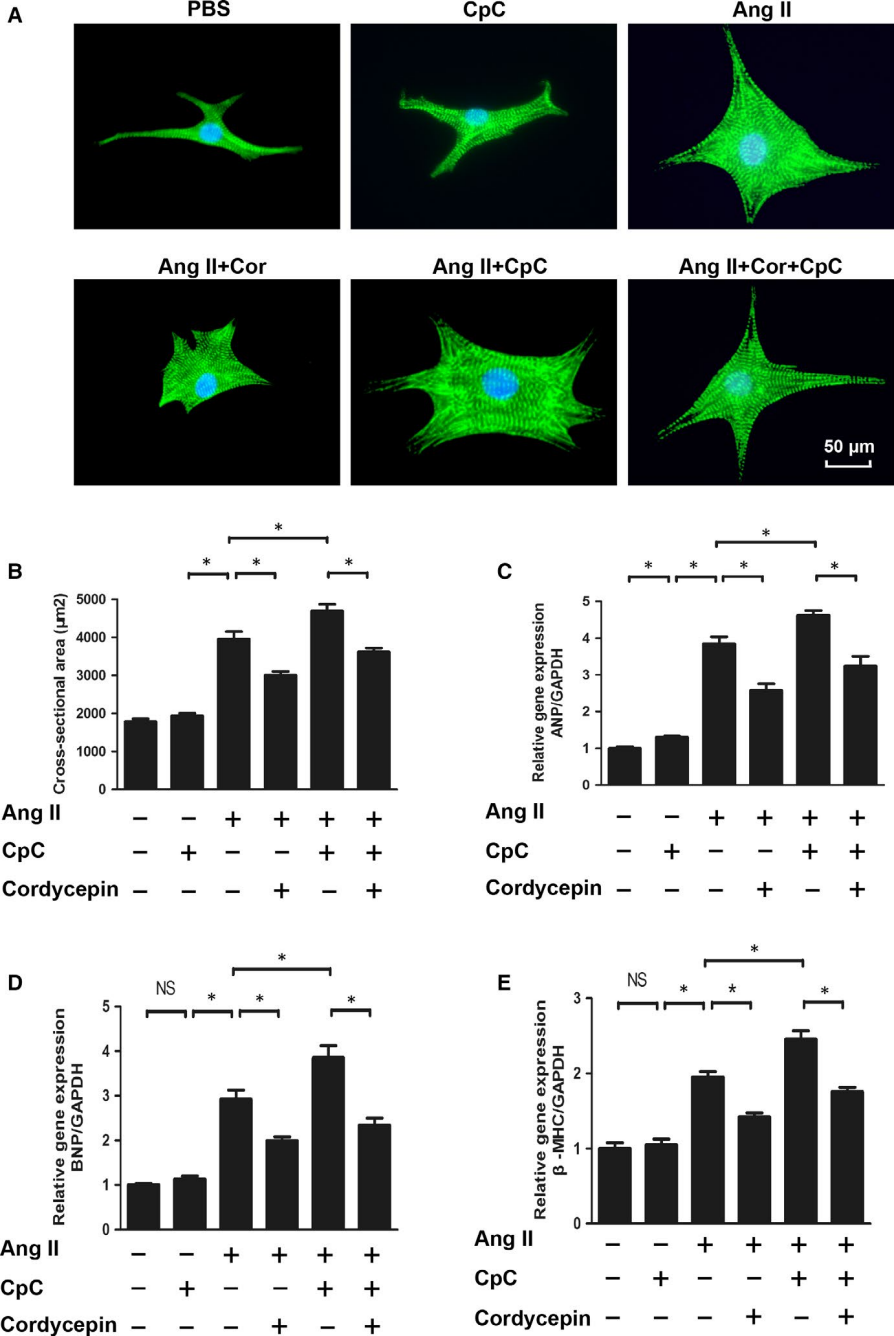
The effects of cordycepin on hypertrophy induced by Ang II were blocked by Compound C (CpC). (A,B) Immunofluorescence staining of a‐actinin and the cell surface area of NRVMs in the indicated groups (n = 6 samples, with 150+ cells per group). (C‐E) The mRNA levels of ANP, BNP, and b‐MHC in NRVMs in each group (n = 6). ^#^ *P* < 0.05 vs the control group; *P < 0.05 vs the Ang II group.

**Figure 6:**
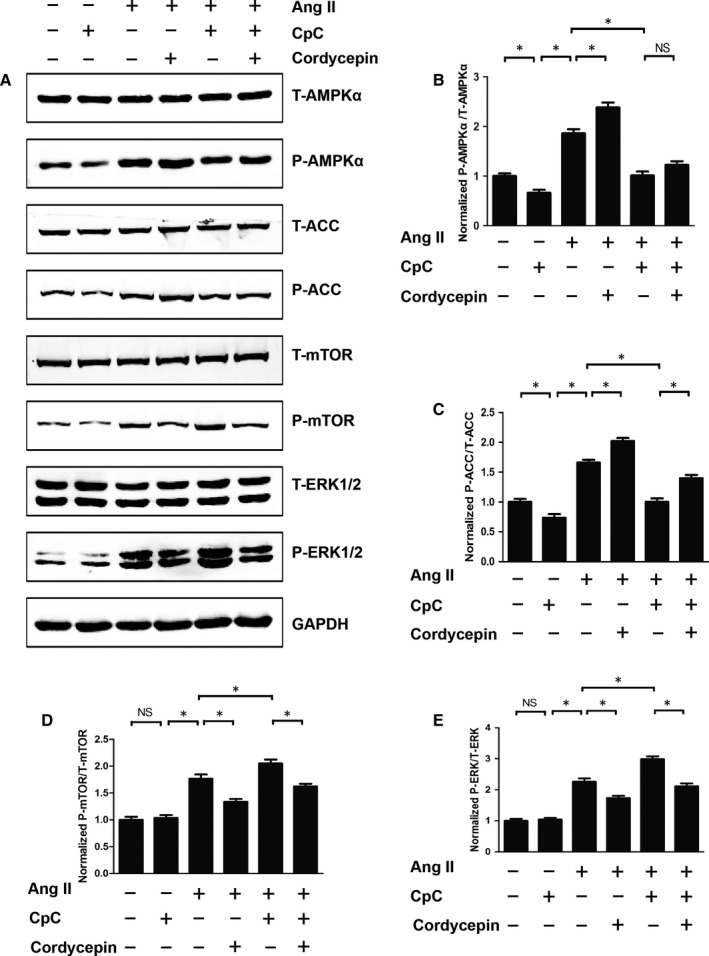
The effects of cordycepin on AMPKα and pro‐hypertrophic pathways were blocked by Compound C (CpC). (A‐E) The protein levels of phosphorylated AMPKα and related targets in indicated groups (n = 6). *P < 0.05 vs the corresponding group

**Figure 7:**
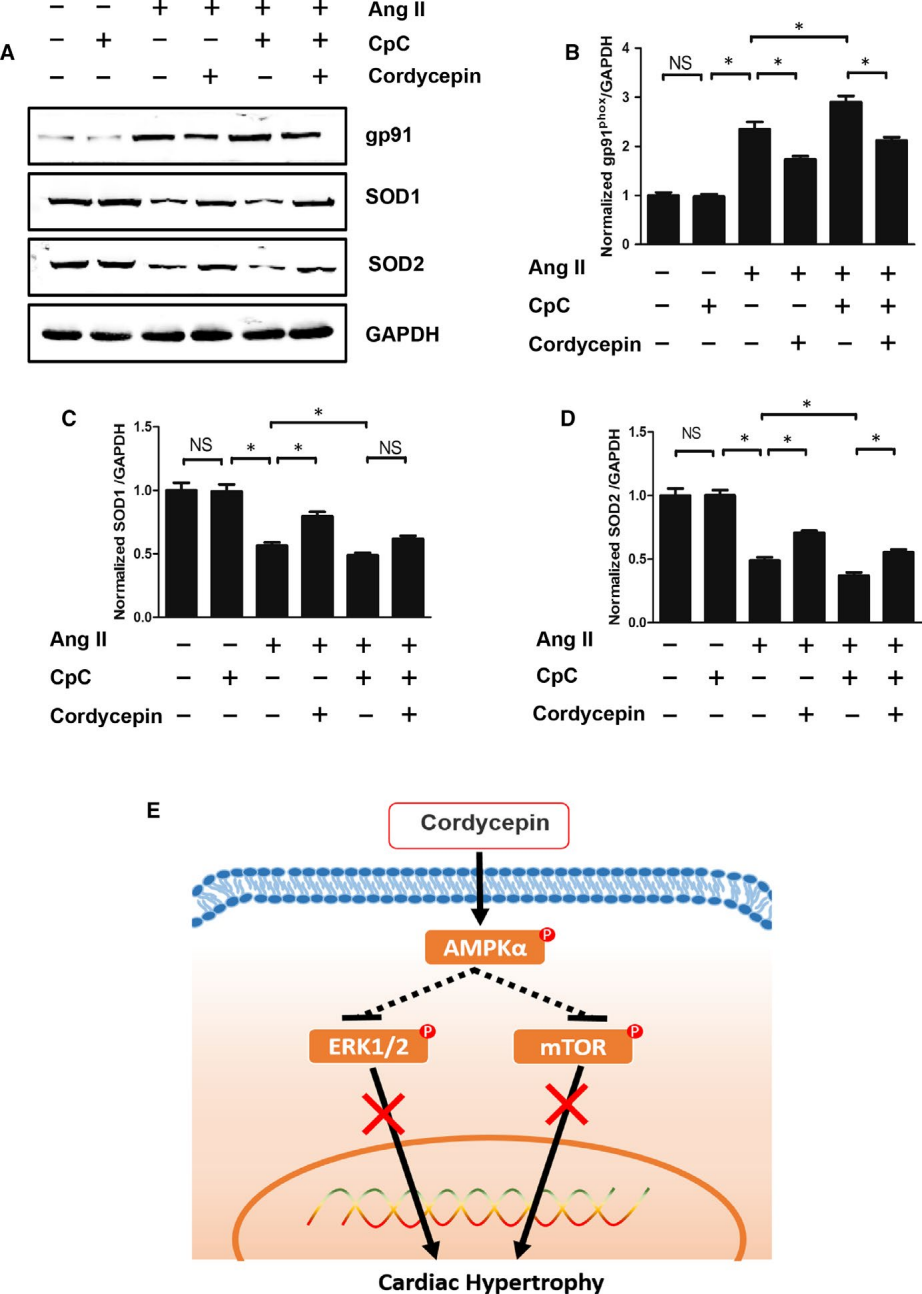
The effects of cordycepin on oxidative stress were blocked by Compound C (CpC) and a mechanistic simulation diagram. (A‐D) The protein levels of gp91^phox^, SOD1, SOD2 in the indicated groups (n = 6). *P < 0.05 vs the corresponding group

To elucidate the anti‐hypertrophic mechanism of cordycepin, NRVMs were treated with 1 μmol/L Ang II in vitro. We next investigated whether NRVMs subjected to CpC treatment showed inhibited the anti‐hypertrophic effect of cordycepin. It was found that in NRVMs stimulated by Ang II, the surface area of NRVMs cells treated with CpC increased significantly and showed an aggravated hypertrophic response, as illustrated by the increased mRNA levels of ANP, BNP and β‐MHC (Figure 5). In addition, we also examined the effects of CpC on AMPKα and downstream signalling pathways. As shown in Figure 6, cordycepin further activated AMPKα and ACC and inhibited the phosphorylation of mTOR and ERK1/2 induced by Ang II in cardiomyocytes. These results were consistent with those of the in vivo experiments. Our study also found that the cordycepin‐mediated activation of AMPKα and inhibition of mTOR and ERK1/2 phosphorylation were abolished by CpC in vitro. In addition, we also detected the protein levels of SOD and gp91^phox^. Ang II significantly reduced the levels of the antioxidant protein SOD and increased the levels of the oxidative stress protein gp91phox, while cordycepin significantly increased SOD levels and decreased gp91^phox^ levels. However, cordycepin lost its inhibitory effects on oxidative stress in NRVMs treated with CpC (Figure 7).

## 4 DISCUSSION

Findings from the current study indicate that cordycepin a novel therapeutic agent attenuated cardiac hypertrophy and improved cardiac function induced by pressure‐overload in vivo and by Ang II in myocytes. Cardiac interstitial fibrosis is one of the main features of cardiac hypertrophy; we also demonstrated that cordycepin can alleviate cardiac fibrosis and accumulation of collagen induced by pressure overload in vivo. The protective effect of cordycepin was mediated by the activation of AMPKα, which inhibited the phosphorylation of mTOR and ERK1/2, as well as by inhibition of oxidative stress injury. Moreover, the protective effects of cordycepin were restrained after AMPKα inhibition by CpC. To our knowledge, our current findings are the first to identify cordycepin as a promising therapeutic agent for cardiac hypertrophy.

Heart failure, the ultimate outcome of various cardiovascular diseases, remains a major cause of morbidity and mortality worldwide. At present, the main strategy for HF treatment involves intervention in HF pathogenesis and subsequent neurohumoral disorders. Despite the increasing number of treatments for HF, patient prognosis remains poor.^5^, ^22^ Cardiac hypertrophy has been identified as a key process contributing to the initiation and progression of HF; this condition mainly involves increased heart weight, altered heart geometry, interstitial fibrosis, increased myocardial cell CSA, and decreased heart function.^23^, ^24^ Over the past decades, clinical and experimental studies have confirmed that oxidative stress (defined as excessive production of ROS relative to antioxidant defence) is increased in HF.^25^ When we studied the protective mechanism of cordycepin against cardiac hypertrophy, we found that cordycepin significantly inhibited oxidative stress associated with hypertrophy.

Cordycepin, the major component isolated from *Cordyceps sinensis* (a well‐known and prized traditional Chinese medicine that is also sold as a health food in many countries)^26^ has been shown to have many pharmacological actions such as antitumour, antibacterial, antioxidant, and anti‐inflammatory effects.^12^, ^26^ Cordycepin is effective for attenuating age‐related oxidative stress and decreasing lipid peroxidation in aged rats.^12^ Cordycepin has also been shown to prevent ischaemia/reperfusion injury in rat hearts via upregulation of haem oxygenase (HO‐1) expression and activation of the Akt/GSK‐3β/p70S6K signalling pathway.^27^ However, our study demonstrated that cordycepin exerted a protective effect against cardiac hypertrophy that was independent of the aforementioned signalling pathway.

The underlying mechanisms by which cordycepin mediates its antihypertrophic effect still remain elusive. Cardiac hypertrophy is a pathophysiological process involving multiple signal transduction pathways and transcription factors.^25^ It has been reported that cordycepin can activate AMPKα (a serine/threonine protein kinase) signalling.^28^, ^29^ AMPKα is an important sensor of cellular energy status, and that regulates this status in various pathophysiological processes.^30^ One of the key characteristics of cardiac hypertrophy is increased protein synthesis and decreased protein degradation in cardiomyocytes, which leads to intracellular protein accumulation. The most important role of AMPKα is to inhibit anabolism and promote catabolism.^10^ A large number of studies have correlated AMPKα with cardiac hypertrophy,^31^ and inactivation of AMPKα promotes cardiomyocyte hypertrophy. In addition, activation of AMPKα by metformin or the AMPKα agonist AICA can inhibit protein synthesis and promote protein degradation in cardiomyocytes.^9^ Because the main subunit of AMPKα in the heart is AMPKα2, gene knockout of AMPKα2 may aggravate cardiac remodelling.^11^ A previous report indicated that cordycepin activates AMPKα via interaction with the γ1 subunit.^29^

Our study clearly demonstrates that AMPKα phosphorylation is significantly increased in both pressure overload‐induced cardiac hypertrophy in vivo and Ang II‐induced cardiac hypertrophy in vitro and that cordycepin further promotes AMPKα phosphorylation. ACC is a downstream protein of AMPKα. Notably, AMPKα phosphorylation promotes ACC phosphorylation.^32^, ^33^ Thus, the levels of phosphorylated ACC reflect the activity of AMPKα. In our experiment, ACC phosphorylation increased during cardiac hypertrophy, and cordycepin further increased ACC phosphorylation more significantly. CpC is an effective, reversible, selective AMPKα inhibitor.^34^, ^35^ In our cell experiments, CpC treatment significantly reduced the phosphorylation of AMPKα and ACC. In addition to ACC, mTOR and ERK1/2 are also downstream targets of AMPKα. Numerous studies have confirmed that mTOR and ERK1/2 are involved in regulating cardiac hypertrophy.^10^, ^36^ Activation of AMPKα can inhibit phosphorylation of mTOR and ERK1/2.^37^ In our study, cordycepin significantly inhibited pressure overload‐ induced mTOR and ERK1/2 hyperphosphorylation, whereas CpC, an AMPKα inhibitor, abolished this inhibition.

The AMPKα signalling system plays a key role in organism and cell survival during metabolic stress and controls mitochondrial function and the redox state.^38^ During the process of cardiac hypertrophy, metabolic dysfunction and oxidative stress damage often occur. Our experiments also confirmed that AB surgery significantly increased the production of gp91^phox^ protein, superoxide generation and MDA and decreased the expression and activity of antioxidant SOD and GSH‐Px. In cell experiments, the addition of the AMPKα inhibitor CpC significantly increased the expression of the oxidative stress protein gp91^phox^ and reduced the expression of the antioxidant protein SOD, which also proves that AMPKα signalling plays an antioxidant role in the process of cardiac hypertrophy. Therefore, this study suggests that cordycepin may play a key role in inhibiting cardiac hypertrophy by activating AMPKα‐related signalling pathways, thereby inhibiting oxidative stress injury in the process of cardiac hypertrophy.

Although we have proven that cordycepin can ameliorate cardiac hypertrophy and have discussed the primary mechanism, our research has some limitations. First, we used CpC, an inhibitor of AMPKα, to conduct cell experiments in vitro, but we have not yet used this compound in animal experiments in vivo. Second, we have not clarified how cordycepin acts on intracellular AMPKα and its downstream signalling pathways. Third, we have not fully elucidated the role of fibrosis. Therefore, in subsequent experiments, we will seek to clarify the detailed protective mechanism of cordycepin.

In conclusion, we have demonstrated that cordycepin prevents cardiac hypertrophy induced by pressure‐overload in vivo and by Ang II in in vitro via by AMPKα. We have also shown that inhibition of AMPKα by CpC abolishes the protective effects of cordycepin against Ang II‐mediated myocyte hypertrophy. Our study provides evidence supporting the use of cordycepin for cardiac hypertrophy treatment. Future research will aim to elucidate the specific mechanism by which cordycepin protects against cardiac hypertrophy, and the potential clinical application of cordycepin will be of great interest.

## CONFLICT OF INTEREST

The authors declare no conflicts of interest.

## Data Availability

The data will be made available after been required upon request from the corresponding author.
