# Supplementary figures and images for: Cordycepin ameliorates cardiac hypertrophy via activating the AMPKα pathway
Source: J Cell Mol Med. 2019 Jun 21;23(8):5715–27. doi: 10.1111/jcmm.14485 (PMC6653598; doi:10.1111/jcmm.14485)

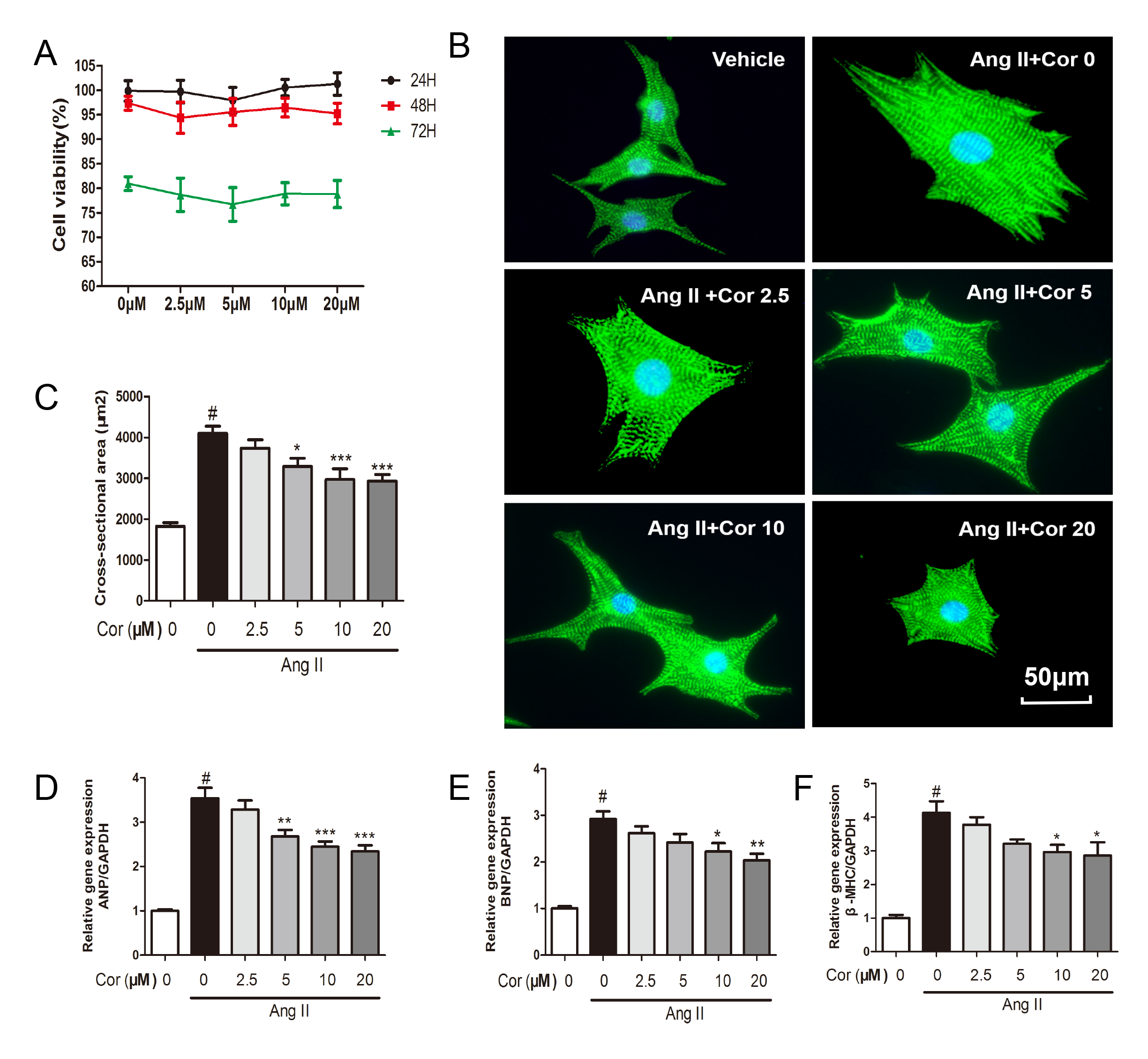

Supplement: Supplementary file 1 [file JCMM-23-5715-s001.tif]
